# 

**Published:** 1926

**Authors:** 


### The Medical Library of the University of Bristol,

WITH WHICH IS INCORPORATED

**The Library of the Bristol Medico-Chirurgical Society.**

*The following donations have been received since the publication of the List in October,* 1926.

December, 1926.

| | |
|---|---|
| Dr. Cecil Clarke (bequest) (1) .. .. .. | 2 volumes |
| Michell Clarke Memorial Fund (2) .. .. | 7 ,, |
| Dr. Kedarnath Das (3) .. .. .. .. | 1 volume |
| Professor E. Fawcett (4).. .. .. .. | 1 ,, |
| Professor E. W. Hey Groves (5) .. .. | 7 volumes |
| Instituto de Anatomia, Lisbon (6) .. .. | 1 volume |
| London School of Tropical Medicine (7) .. | 1 ,, |
| Professor C. Lloyd Morgan (8) .. .. .. | 1 ,, |
| Dr. G. Parker (9) .. .. .. .. | 1 ,, |
| Rockefeller Foundation (10) .. .. .. | 1 ,, |

Unbound periodicals have also been received from Professor Hey Groves, Dr. J. O. Symes, and Dr. Carey F. Coombs.

### THE ONE HUNDRED AND EIGHTEENTH LIST OF BOOKS.

The figures in round brackets refer to the figures after the names of the donors. The books to which no such figures are attached have either been bought from the Library Fund or received through the *Journal.*

**Bainbridge, W. S.** .. *Second International Congress on Military Medicine and Pharmacy* .. .. .. .. .. .. .. .. (5) 1925

**Ballance, C. A.** .. .. *Surgery of Temporal Bone.* 2 Vols. .. .. (2) 1919

### Library.

| | | | | |
|---|---|---|---|---|
| **Baronaki, Dr.** | *Les néoplasmes et leur thérapeutique médicale* | | | 1927 |
| **Bernhard, O.** | *Light Treatment in Surgery* | | | 1926 |
| **Broderick, R. A.** | *Dental Bacteriology* | | | 1926 |
| **Burnham, A. C.** | *First Aid and Emergency Treatment* | | (5) | 1917 |
| **Cathcart, G. C.** | *Treatment of Chronic Deafness* | | | 1926 |
| **Cheesman, J. E.** | *Baillière's Synthetic Anatomy.* Parts I.–III. | | | 1926 |
| **Clark, C. A.** | *Dental Radiography* | | | 1926 |
| **Dally, J. F. H.** | *High Blood Pressure* | 2nd Ed. | | 1926 |
| **Darling, H. C. R.** | *Surgical Nursing and After Treatment* | 2nd Ed. | (5) | 1923 |
| **Das, K.** | *Textbook of Midwifery* | | (3) | 1921 |
| **"Doctor Robin"** | *What It Feels Like* | | | 1926 |
| **Ewart, E. D.** | *Guide to Anatomy* | 2nd Ed. | | 1926 |
| **Ferguson, J. B.** | *Quartz Mercury Vapour Lamp* | | | 1926 |
| **Fitzwilliams, D. C. L.** | *A Pocket Surgery* | | (5) | 1921 |
| **Gabell, G.** | *Prosthetic Dentistry* | | | 1921 |
| **Hutchison, R., and Rainy, H.** | *Clinical Methods* | 6th Ed. | (1) | 1918 |
| **Kennedy, A. M.** | *Medical Case Taking* | | | 1926 |
| **Labat, G.** | *Regional Anæsthesia* | | (2) | 1924 |
| **Levin, O. L.** | *Your Hair and Your Health* | | | 1926 |
| **Loeb, L.** | *Endema* | | (9) | 1923 |
| **Logan, J. D.** | *Dental Prosthetics* | | | 1926 |
| **Malcolm-Smith, G. L.** | *Hewat's Examination of the Urine* | 7th Ed. | | 1926 |
| **Marsden, P. H.** | *Dental Materia Medica* | | | 1926 |
| **Meek, A.** | *Essentials of Zoology* | | (8) | 1922 |
| **Miller, E.** | *Types of Mind and Body* | | | 1926 |
| **Moynihan, B.** | *Abdominal Operations.* 2 Vols. | 4th Ed. | (2) | 1926 |
| **Naito, I.** | *Die hyperostosen des schadels* | | (5) | 1924 |
| **Parsons, J. H.** | *Diseases of the Eye* | 5th Ed. | (2) | 1926 |
| *The Practitioner's Guide to Clinical Research* | | 2nd Ed. | (5) | 1921 |
| *Radium Treatment of Cancer of Uterus* (Cancer Research Committee) | | | | 1926 |
| **Razes** | *Traité sur le calcul dans les reins et dans la vessie* | | | 1896 |
| **Redding, J. M.** | *X-ray Diagnosis* | | | 1926 |
| **Schmieden and Turnbull.** | *The Course of Operative Surgery* | 2nd Ed. | (5) | 1920 |
| **Sequeira, J. H.** | *Diseases of the Skin* | 4th Ed. | (2) | 1927 |
| **Smith, G. F. R.** | *Dental Anæsthesia* | | | 1926 |
| **Ten Teachers** | *Diseases of Women (Ed. Comyns Berkeley)* | 3rd Ed. | (2) | 1926 |
| **Watson, J. H.** | *Fundamentals of the Art of Surgery* | | | 1926 |
| **White, W. H.** | *Materia Medica, Pharmacy, Pharmacology* | 8th Ed. | (1) | 1903 |
| **Woodwark, A. S.** | *Manual of Medicine* | 2nd Ed. | (2) | 1920 |

### TRANSACTIONS, REPORTS, JOURNALS, ETC.

| | | |
|---|---|---|
| Anatomischer Anzeiger. Bds. I. (1886) and XIII. (1897) | | |
| Archivio de Anatomia e Antropologia. Vol. VIII. | (6) | 1923 |
| Collected Essays and Laboratory Studies. Vol. II. | (7) | 1925–6 |
| Congrès Francais de Médicine. XVIII<sup>e</sup> Session | | 1925 |
| Medical Society's Transactions. Vol. XLVIII. | | 1925 |
| Methods and Problems of Medical Education. 5th Series | (10) | 1926 |

Progressive Medicine. Vol. III. .. .. .. .. .. .. .. .. .. .. .. 1926
Published Papers by the Staff of the Middlesex Hospital .. .. (4) 1925–6
Reports of St. Andrew's Institute. Vol. III. .. .. .. .. .. .. .. .. 1926
Surgical Clinics of North America. Vol. VI. No. 5 .. .. .. .. .. .. 1926
Transactions of the College of Physicians of Philadelphia. Vol. XLVII. 1925

### Local Medical Notes.

### EXAMINATION RESULTS.

**University of Bristol.**—Students of the University have recently passed the following examinations :—

M.B., Ch.B.—*Supplementary First Examination. Pass :* L. P. Ashton, J. E. Brown, K. P. Beaubrun, G. E. Chadd, J. L. S. Coulter, D. Dewe, R. H. Moore, E. S. G. Owen, J. G. Watts. *Pass in Physics and Biology only :* W. R. Cambridge. *Pass in Chemistry and Biology only :* C. J. N. Davis, C. N. Royal.

M.B., Ch.B. (*Part II.*).—*Second Examination. Pass :* J. L. W. Davies.

Diploma in Dental Surgery.—*Supplementary Preliminary Science Examination for Dental Students. Pass : In Biology only* (*Completing Examination*) *:* T. A. A. Eaves, K. V. M. Taylor.

L.D.S.—*First Professional Examination. Pass. In Anatomy and Physiology* (*Completing Examination*) *:* C. R. H. Edwards, L. W. G. Jones. *In Anatomy and Physiology :* (Miss) B. J. Shapland.

L.D.S., R.C.S.—*First Professional Examination. Part I.* (*Dental Mechanics*) *:* R. G. Boodle. *Part III.* (*Anatomy and Physiology*) *:* R. G. Boodle, C. Haskins, (Miss) M. E. Minton, R. A. Phillips, J. P. Price, L. Symonds.

### APPOINTMENTS.

Bristol Royal Infirmary.—Honorary Surgeon : H. Chitty, M.D., M.S. Lond., F.R.C.S. Assistant Physician in charge of Ante - Natal Department : Lily A. Baker, B.A., M.B., B.Ch. Dubl., F.R.C.S.I. Honorary Obstetrical Registrar : H. L. Shepherd, M.B., Ch.M. Bristol.

### Index.

Adams, A. W.—The Treatment of Congenital Club Foot, 232.
Annual Medical Dinner, 230.
Annual Service for the Medical Profession, 49.
Autonomic Nervous System, Relation of to Clinical Medicine.—J. R. Charles, 127.

Bodman, J. Hervey, and Rogers, Beatrice.—Polyneuritis, 84.
Books, Reviews of—
- Abdomen, Early Diagnosis of the Acute.—Z. Cope, 39.
- Anatomy, A Guide to: for Students of Medical Gymnastics, Massage and Medical Electricity.—E. D. Ewart, 218.
- Aphasia.—S. A. Kinnier Wilson, 108.
- Beattie, J. M. and Dickson, W. E. C. —A Text-book of Pathology, 45.
- Bennett, T. I.—The Stomach and Alimentary Canal in Health and Disease, 43.
- Bland-Sutton, Sir John, and Giles, A. E.—The Diseases of Women, 224.
- Brain and Heart.—G. Fano, 174.
- Brockbank, E. M.—Incapacity or Disablement in its Medical Aspects. 111.
- Burridge, H. A.—An Introduction to Forensic Medicine, 118.
- Cameron, H. C.—Diseases of Children, 220.
- Cathcart, G. C.—Hunter Tod's Diseases of the Ear, 222.
- Cathcart, G. C.—Treatment of Chronic Deafness by the Electrophonoide, 223.
- Children, Diseases of. — H. C. Cameron, 220.
- Chronic Deafness by the Electrophonoide, Treatment of.—G. C. Cathcart, 223.
- Coaching Manual for Midwifery Students.—Félice Norton, 39.
- Colwell, H. A. and Wakely, C. P. G.—Introduction to the Study of X-rays and Radium, 115.
- Cope, Z.—Early diagnosis of the Acute Abdomen, 39.

Books, Reviews of (continued)—
- Crookshank, F. G.—Migraine and other Common Neuroses, 110.
- Cumberbatch, E. P. and Robinson, C. A.—Treatment of Gonococcal Infection by Diathermy, 116.
- Dickson, I. Wanless.—Rational Gland Therapy for Women, 114.
- Diet, Invalid.—Dorothy Morton, 226.
- "Doctor Robin."—What it Feels Like, 217.
- Ear, Hunter Tod's Diseases of the.—G. C. Cathcart, 222.
- Easterbrook, C. C.—Mental Invalids, 109.
- Ewart, E. D.—A Guide to Anatomy: for Students of Medical Gymnastics, Massage and Medical Electricity, 218.
- Extra Pharmacopœia, The.—W. H. Martindale and W. W. Westcott, 41.
- Fano, G.—Brain and Heart, 174.
- Fisher, A. G. Timbrell.—Manipulative Surgery, 38.
- Ferguson, J. Bell.—The Quartz Mercury Vapour Lamp, 225.
- Forensic Medicine, An Introduction to.—H. A. Burridge, 118.
- Fox, E. Margaret.—First Steps in Nursing, 226.
- Fractures and Dislocations in General Practice, Treatment of.—M. Page and W. R. Bristow, 113.
- Fraser, J.—Surgery of Childhood, 174.
- Gibson, A. G.—The Heart, 220.
- Girdlestone, G. R.—Diagnosis and Treatment of Tuberculosis of the Hip, 38.
- Gland Therapy for Women, Rational. —I. Wanless Dickson, 114.
- Gonococcal Infection, Treatment of by Diathermy.—E. P. Cumberbatch and C. A. Robinson, 116.
- Gould's Medical Dictionary.—R. J. E. Scott (Ed.), 217.
- Gray, A.—A Synopsis of Gynæcology, 39.
- Gynæcology, A Synopsis of.—A. Gray, 39.
- Heatherley, Francis. — Modern Methods in Heart Disease, 220.

### INDEX.

BOOKS, REVIEWS OF (continued)—

Heart Affections, The Principles of Diagnosis and Treatment in.—James Mackenzie and James Orr, 219.

Heart, Disease of.—Sir J. Mackenzie, 108.

Heart Disease, Modern Methods in.—Francis Heatherley, 220.

Heart, The.—A. G. Gibson, 220.

Hernaman - Johnson, F.— Radiotherapy in Relation to General Medicine, 175.

History of Medicine.—M. Neuburger, 45.

Hornibrook, F. A.—Physical Fitness in Middle Life, 112.

Hutchison, R. (Ed.).—An Index of Treatment, 43.

Ibbotson, W.—Surgical Operations, 221.

Incapacity or Disablement in its Medical Aspects.—E. M. Brockbank, 111.

Index of Treatment, An. — R. Hutchison (Ed.), 43.

Kenwood, H. R.—Public Health Laboratory Work, 40.

Labour, The Abdomen in.—R. N. Porritt, 224.

Lamp, The Quartz Mercury Vapour —J. Bell Ferguson, 225.

Mackenzie, James, and Orr, James.—The Principles of Diagnosis and Treatment in Heart Affections, 219.

Mackenzie, Sir J.—Diseases of the Heart, 108; The Basis of Vital Activity, 116.

Manipulative Surgery.—A. G. Timbrell Fisher, 38.

Martindale, W. H. and Westcott, W. W.—The Extra Pharmacopœia, 41.

Medical Annual, The, 176.

Medical Electricity and Radiology, Handbook of.—J. R. Riddell, 225.

Mental Invalids.—C. C. Easterbrook, 109.

Migraine and other Neuroses.—F. G. Crookshank, 110.

Morison, R. and Saint, C.—An Introduction to Surgery, 112.

Morton, Dorothy.—Invalid Diet, 226.

Murrell, W.—What to do in Cases of Poisoning, 117.

Nervous System, Diseases of the.—H. C. Thomson and G. Riddoch, 117.

Neuburger, M.—History of Medicine, 45.

Norton, Félice.—Coaching Manual for Midwifery Students, 39.

BOOKS, REVIEWS OF (continued)—

Nurses, A Handbook for.—J. K. Watson, 226.

Nursing, First Steps in.—E. Margaret Fox, 226.

Obesity.—Leonard Williams, 221.

Operations, Surgical.—W. Ibbotson, 221.

Operative Orthopædics.—A. Steindler, 113.

Page, C. M. and Bristow, W. R.—Treatment of Fractures and Dislocations, 113.

Pathology, General and Special.—J. M. Beattie and W. E. C. Dickson, 45.

Paul, F. T.—Selected Papers, Surgical and Pathological, 44.

Perkins, G.—Tuberculous Disease of the Hip Joint, 175.

Physical Fitness in Middle Life.—F. Hornibrook, 112.

Poisoning, What to do in Cases of.—W. Murrell, 117.

Porritt, R. N.—The Abdomen in Labour. 224.

Postgraduate Medical Journal, The, 42.

Post-mortem Appearances.—Joan M. Ross, 42.

Practice of Medicine, Text-book of.—F. W. Price (Ed.), 116.

Prescriptions, Favourite.—E. Ward, 177.

Price, F. W. (Ed.).—A Text-book of the Practice of Medicine, 116.

Public Health Laboratory Work.—H. R. Kenwood, 40.

Radiotherapy in Relation to General Medicine.—F. Hernaman-Johnson, 175.

Riddell, J. R.—Handbook of Medical Electricity and Radiology, 225.

Ross, Joan M. — Post - mortem Appearances, 42.

Scott, R. J. E. (Ed.).—Gould's Medical Dictionary, 217.

Selected Papers, Surgical and Pathological.—F. T. Paul, 44.

St. Andrew's (James Mackenzie) Institute for Clinical Research, St. Andrew's, Fife, Reports of the —David Waterston (Ed.), 219.

Steindler, A.—Operative Orthopædics, 113.

Stomach and Alimentary Canal, The. —T. I. Bennett, 43.

Surgery, An Introduction to.—R. Morison and C. Saint, 112.

Surgery of Childhood.—J. Fraser, 174.

Thomson, H. C. and Riddoch, G.—Diseases of the Nervous System, 117.

### Index.

Books, Reviews of (continued)—
Tuberculous Disease of the Hip Joint. —G. Perkins, 175.
Tuberculosis of the Hip, Diagnosis and Treatment of. — G. R. Girdlestone, 38.
Vital Activity, The Basis of.—Sir J. Mackenzie, 116.
Ward, E.—Favourite Prescriptions, 177.
Waterston, David (Ed.).—Reports of the St. Andrew's (James Mackenzie) Institute for Clinical Research, St. Andrew's Fife, 219.
Watson, J. K.—A Handbook for Nurses, 226.
What it Feels Like. — "Doctor Robin," 217.
Williams, Leonard.—Obesity, 221.
Wilson, S. A. Kinnier.—Aphasia, 108.
Women, The Diseases of.—Sir John Bland-Sutton and A. E. Giles, 224.
X-Rays and Radium.—H. A. Colwell and C. P. G. Wakely, 115.
Bristol Medico-Chirurgical Journal, 47, 227.

Cardiac Disease, Ætiology of.—Carey F. Coombs, 1.
Carleton, H. H.—Some Problems of Pulmonary Disease, 54.
Cervical Erosion, The Pathology of.—G. Hadfield, 151; Treatment of.—H. J. D. Smythe, 156.
Chambers, E. R.—The Ocular Signs of Migraine, 29.
Charles, J. R.—Relation of Autonomic Nervous System to Clinical Medicine, 127.
Coathupe, E. W.—Obituary, 238.
Congenital Club Foot.—A. W. Adams, 232.
Connellan, P. S.—Treatment of Repressed Memories by Hypnotism, 209.
Coombs, Carey F.—The Ætiology of Cardiac Disease, 1.

Discussions :
The Ocular Signs of Migraine.—E. R. Chambers. Discussed by A. E. J. Lister, 36; J. R. Charles, 37; H. H. Carleton, 37.
The Treatment of Congenital Club Foot.—A. W. Adams. Discussed by E. W. Hey Groves, 234; H. Chitty, 234; 234; E. Watson-Williams, 234; W. A. Jackman, 234; A. L. Flemming, 235.
Uveo-Parotitic Paralysis.—G. Parker. Discussed by Carey F. Coombs, 82; A. E. J. Lister, 82; P. Phillips, 82.

Editorial Committee, 227.

Faraker, Erskine.—Kala azar, 183.
Fawcett, E., Hadfield, G., and Phillips, P.—Suprarenal Virilism, 20.

Gillies, H. D.—Plastic Surgery, 122.

Hadfield, G.—Pathology of Cervical Erosion, 151.
Hadfield, G., Fawcett, E., and Phillips, P.—Suprarenal Virilism, 20.
Herpes Zoster and Varicella, A Case of. —R. E. Overton, 169.
Hospital for Bristol Cripples, 178.
Hospital Sunday, 48.

Iles, A. E.—Treatment of Lachrymal Obstruction, 191.
Insulin in Surgery, The Use of.—J. A. Nixon, 199.

Kala azar.—Erskine Faraker, 183.

Lachrymal Obstruction.—A. E. Iles, 191.
Local Medical Notes, 64, 126, 182, 242.
Long Fox Memorial Lecture.—Carey F. Coombs, 1, 51.

Medical Dinner, 230.
Medical Library of the University of Bristol, 62, 124, 180, 240.
Meetings of Societies—
Bristol Medico-Chirurgical Society, 53, 122, 231.
British Medical Association, Bath and Bristol Branch, 58.
Meningitis, Operative Treatment of.—E. Watson-Williams, 91.
Morgan, C. Lloyd.—Personality: A Review, 104.

Nixon, J. A.—The Use of Insulin in Surgery, 199; Rigid Rest in Rheumatic Carditis, 96.

Obituary—
A. W. Prichard, 59; C. K. Rudge, 235; E. W. Coathupe, 238.
Ocular Signs of Migraine, The.—E. R. Chambers, 29.
Overton, R. E.—A Case of Severe Herpes Zoster and Varicella, 169.

Parker, G.—Uveo-Parotitic Paralysis, 73.
Personality: A Review.—C. Lloyd Morgan, 104.
Phillips, P., Fawcett, E., and Hadfield, G.—Suprarenal Virilism, 20.

### Index.

Plastic Surgery, Functional and Æsthetic Results of.—H. D. Gillies, 122.
Polyneuritis, Preceded by Inflammation of the Parotid, Lachrymal and Mammary Glands.—Beatrice Rogers and J. H. Bodman, 84.
Prichard, A. W.—Obituary, 59.
Pulmonary Disease, Some Problems of. —H. H. Carleton, 54.

Queen Alexandra Memorial Hospital, Weston-super-Mare, 228.

Renal Disease.—D. G. C. Tasker, 161.
Repressed Memories, Treatment of by Hypnotism.—P. S. Connellan, 209.
Rheumatic Carditis, Rigid Rest in.—J. A. Nixon, 96.
Rogers, Beatrice, and Bodman, J. Hervey.—Polyneuritis, 84.
Royal Institute of Public Health, 120.
Rudge, C. King.—Obituary, 235.

Smythe, H. J. D.—Treatment of Cervical Erosion, 156.
Suprarenal Virilism.—E. Fawcett, G Hadfield and P. Phillips, 20.

Tasker, D. G. C.—Renal Disease, 161.

University of Strasbourg, 121.
Uveo-Parotitic Paralysis.—G. Parker, 73.

Watson-Williams, E.—Operative Treatment of Meningitis, 91.
Watson-Williams, P., Resignation of Editorship, 119.
Wellcome Historical Medical Museum, 229.
Wills, Sir George, 48, 227.
Wright, J., and Sons, Centenary, 52.

